# Promoting Local Development and Food Literacy in a Rural Angolan Community

**DOI:** 10.3390/nu17172788

**Published:** 2025-08-28

**Authors:** Sofia Campos, Joana Andrade, Eduardo Santos, Inês Figueiredo, Vitor Martins, Eugénia Matos, Ana Paula Cardoso, Manuela Ferreira

**Affiliations:** 1 Health Sciences Research Unit: Nursing (UICISA: E), Nursing School of Coimbra (ESEnfC), Health School of the Polytechnic Institute of Viseu, 3504-510 Viseu, Portugal; ejf.santos87@gmail.com; 2 Unidade Local de Saúde de Viseu Dão-Lafões, E.P.E, 3504-509 Viseu, Portugal; joanaandrade@saudeportugues.org (J.A.); inesfigueiredo@saudeportugues.org (I.F.); vitormartins@saudeportugues.org (V.M.); 3 Unidade Local de Saúde de Santa Maria, 1649-028 Lisboa, Portugal; emmmpf@gmail.com; 4 Higher School of Education of Viseu, Ci&DEI, Polytechnic Institute of Viseu, 3504-510 Viseu, Portugal; a.p.cardoso@esev.ipv.pt

**Keywords:** food knowledge, health professionals, training model

## Abstract

Background/Objectives: In Angola, malnutrition contributes each year to the deaths of an estimated 42,000 to 76,000 children under the age of 5. Addressing this issue must stand as a priority and requires providing local residents with access not only to nutritious food but also to adequate and accurate information in order to facilitate informed dietary choices. As part of the “Seigungo—Health, Education and Quality of Maternal and Child Life in Gungo project”, a nutrition-focused study was conducted in Gungo, Angola to evaluate the effectiveness of a training model designed to enhance food literacy among residents. Methods: Data were collected using a 14-item questionnaire developed to assess various key domains of food literacy: information seeking and access; comprehension and thematic knowledge; critical evaluation of information and behaviour; practical application and sound decision-making. Results: Thirty trainees took part in the study, of which 60% were men, with a mean age of 45.6 years. The majority were single (53.3%) and had completed six years of formal education (26.7%). Before attending the training program, 86.7% of the participants demonstrated inadequate or problematic food literacy. Following the intervention, the proportion of participants with adequate food literacy increased significantly from 13.3% to 73.3% (*p* < 0.001). Conclusions: The training program had a statistically significant impact on improving food literacy.

## 1. Introduction

Complementary feeding is fundamental for healthy growth and development during childhood, particularly within the first thousand days of life, a period widely recognised as critical for both short-term and long-term health outcomes [1]. The adequate introduction of solid foods alongside continued breastfeeding is crucial to ensure the intake of macro- and micronutrients required for a child’s physical, cognitive, and immunological development [2]. The World Health Organization (WHO) recommends introducing complementary feeding at around six months of age, while maintaining breastfeeding until at least two years of age or beyond, whenever possible [1]. However, despite the existence of well-defined international guidelines and recommendations, the existing literature suggests that the implementation of these practices varies significantly depending on socio-economic, cultural, and geographical contexts [3]. In a multicentre study that included health professionals from 46 countries, the authors identified different patterns for complementary diets: in Asia and Africa, for instance, grains are the most commonly used complementary infant foods, whereas vegetables are the most common in Europe and America. This variation reflects not only differences in food availability but also the impact of cultural traditions and existing public health policies [3]. It is therefore clear that infant feeding guidelines must be adapted to local realities and take into consideration critical aspects such as available resources, cultural habits, and the health literacy of the different communities. This need becomes even more urgent in contexts affected by structural poverty, such as many regions of Angola. National data indicate that the prevalence of malnutrition among Angolan children is extremely high: approximately 38% of children under five are moderately malnourished, while 15% suffer from severe malnutrition [4]. In rural areas, particularly in southern Angola, the situation is particularly alarming, with acute malnutrition rates exceeding 11% [4].

A study by Mekonen et al. [5] in sub-Saharan Africa identified that only 13% of children between 6 and 23 months had adequate complementary feeding practices, with maternal education, use of health services, and dietary diversity being the main determinants of this behaviour. Research at the Missionary Catholic Hospital of Chiulo situated in Cunene province, provides robust empirical data on the risk factors and prognosis of SAM among hospitalised children. In that study, the authors identified several factors directly associated with severe acute malnutrition: early suspension of breastfeeding, inadequate introduction of complementary foods, low dietary diversity, and limited caregiver knowledge of the child’s nutritional needs [6]. Their research also shows that, despite the existence of food in many households, it was often inadequately prepared or unevenly distributed, which suggests the presence of educational and cultural barriers.

Promoting food literacy through nutrition education campaigns specifically tailored to the local context represents an effective and low-cost strategy for preventing child malnutrition [6]. Vidgen et al. define food literacy as “a set of interrelated knowledge, skills, and behaviours that enable individuals to plan, manage, select, prepare, and consume food to meet their needs and influence their dietary intake” [7]. This definition emphasises the multifaceted nature of food literacy, which ranges from practical skills, such as cooking and interpreting nutrition labels, to critical skills, such as assessing food quality and understanding the health and environmental impact of food choices. Truman et al. broaden this perspective by identifying six core domains of food literacy: skills and behaviours, food/health choices, culture, knowledge, emotions, and food systems [8]. This approach recognises that food decisions are influenced by a wide range of factors, including cultural traditions, emotional associations with food, and the structure of food systems.

In African contexts, food literacy is particularly important due to the challenges associated with food security, nutritional transitions, and changes in food systems. In their study, Fisher et al. [9] developed a definition of food literacy adapted to the South African context that highlights the impact of an individual’s knowledge, skills, and behaviours on the sourcing, selection, preparation, and consumption of food [9]. This definition also emphasises the nutritional, economic, safety, and social aspects of food. To address the need for culturally sensitive instruments capable of assessing and promoting food literacy, a food literacy scale for adults was developed and validated for East African countries, specifically Uganda and Kenya. This study underscores the importance of understanding and promoting food literacy in a context-specific manner, consistently accounting for the cultural, social, and economic particularities of each region.

Against this backdrop, the Seigungo—Health, Education and Quality of Maternal and Child Life in Gungo project was implemented in a rural community in Kwanza Sul province, Angola. Gungo, a poverty-stricken, mountainous community reliant on small-scale agriculture and fishing, faces chronic food insecurity due to erratic rainfall, limited resources, and a lack of healthcare professionals. A preliminary assessment of 458 children, conducted for our team, revealed that 73.6% were underweight, 46% were malnourished, and 19% were severely malnourished. This problem led us to question how health promoters empower populations to use available food resources. Considering the findings of the diagnostic assessment, we hypothesise that promoting health literacy could be an effective strategy to address child malnutrition in vulnerable contexts, such as the Gungo commune.

## 2. Materials and Methods

### 2.1. Study Design and Participants

The study is part of the research project “Seigungo—Health, Education and Quality of Maternal and Child Life in Gungo”, focusing specifically on food literacy. This was a before-and-after study that employed descriptive-correlational analysis. The research was conducted in strict accordance with the Strengthening the Reporting of Observational Studies in Epidemiology (STROBE) [10] guidelines that ensure methodological rigour in the reporting of observational studies. The research was conducted in Gungo commune, located in the city of Sumbe, Kwanza Sul province, Angola. This region has an estimated population of 33,969 inhabitants, distributed across approximately 108 districts. Poverty levels are extremely high and affect over 80% of the population.

### 2.2. Training Program

The training program offered was specifically designed for these community agents, who are also the primary focus of this research. Before the intervention, a diagnostic evaluation was carried out. The training program included 30 trainees.

The inclusion criteria for participants were being over 18 years of age, being able to read and write, and working as a health promoter in Gungo. These promoters were identified and recruited through contact with local authorities in the commune. This program was divided into two stages: an initial screening in October 2023, followed by theoretical and practical instruction in January 2024 and post-training data collection in October 2024. The training intervention in the area of nutrition addressed topics such as breastfeeding, formula feeding, dietary diversification, vitamin supplementation, food intolerances and allergies, adequate nutrition, nutrition up to the age of 6, considering the cultural context, and accessible foods in Gungo (responsive nutrition).

### 2.3. Ethical Considerations

All ethical and deontological procedures were observed, and the study and project obtained a favourable opinion on the 24 April 2025 from the Ethics Committee of the Polytechnic Institute of Viseu, Portugal (Reference No. 19/SUB/2025). The study follows the principles of the Declaration of Helsinki. The ethics report was issued after data collection because Gungo does not have an ethics committee, and it was necessary to adhere to the schedule agreed upon with the Portuguese trainers. This made rescheduling impossible. The Polytechnic Institute of Kwanza Sul acted as the Angolan partner for the study. All participants were informed and given explanations of the meaning of informed consent. Consent was written into the survey, although due to sociolinguistic restrictions, a clear and culturally adapted oral explanation was provided to ensure full understanding. Participation was completely voluntary, and the anonymity and privacy of the data collected were protected. All ethical standards were maintained to ensure the protection of participants and the integrity of the study, resulting in approval by the ethics committee.

### 2.4. Statistical Analysis

A specific sociodemographic questionnaire was developed to characterise the sample (e.g., sex, age, marital status, level of education, years of work experience, displacement from usual residence and household). Simultaneously, a 14-item instrument was designed and administered to assess various key domains: information search and access; understanding and thematic knowledge; critical evaluation of information and behaviour; and the practical application of knowledge in making informed decisions. The participants were instructed to answer the following questions using a five-point scale: 1 = Very easy; 2 = Easy; 3 = Difficult; 4 = Very difficult; and 5 = Don’t know/No answer. (Table 1).

For the score’s operationalisation, the 14 items should be dichotomised: answers marked ‘difficult’ and ‘very difficult’ should be assigned a value of 0, while answers marked ‘easy’ and ‘very easy’ should be assigned a value of 1. The total score is obtained by summing the values of all 14 items and reflects the level of food literacy of each participant. A score of 13 or above indicates ‘adequate’ food literacy, scores between 9 and 12 indicate ‘problematic’ food literacy and scores of 8 or below indicate ‘inadequate’ food literacy. This instrument demonstrated strong internal consistency, with a Cronbach’s alpha coefficient of 0.83 for the overall scale.

Statistical analysis was conducted using IBM^®^ SPSS^®^ Statistics software, version 29.0, IBM Corp., Armonk, NY, USA. A descriptive analysis of the data was performed to calculate absolute frequencies (n), percentages (%), measures of central tendency (mean—M), and the measure of dispersion (standard deviation—SD). The reliability of the instrument was assessed using Cronbach’s alpha coefficient (α), and the following threshold values were established: >0.9 (excellent); between 0.8 and 0.9 (good); between 0.7 and 0.8 (acceptable); between 0.6 and 0.7 (questionable); between 0.5 and 0.6 (poor); and <0.5 (unacceptable).

The choice of the statistical techniques was determined by the nature and characteristics of the variables under analysis. Prior to any analysis, the assumptions of normality were confirmed. The skewness and kurtosis values, as well as the value of the Shapiro–Wilk test statistic and its *p*-value, showed that the data did not follow a normal distribution (*p* < 0.05). Non-parametric tests, namely the Wilcoxon test, were used for inferential analysis to compare the means of a quantitative variable in paired groups across two observation points. In addition, to assess whether there were differences between the participants after completing the training programme in terms of gender, marital status, age, and years of work experience, the Mann–Whitney U test, the Kruskal–Wallis H test and Spearman’s rank correlation coefficient were used, respectively. A value of *p* < 0.05 was considered statistically significant.

## 3. Results

The sample consisted of 30 participants with a mean age of 45.5 ± 10.9 years, predominantly male, single, and having completed 6 years of formal education. The Angolan education system is divided into primary education (6 years), secondary education I cycle (3 years), secondary education II cycle (4 years), and higher education. Most of the respondents reported being displaced from their usual place of residence and were living with other family members. Participants had an average of 13.8 ± 8.5 years of work experience (Table 2).

### Implementation of the Training Program

Before their participation in the training program, 86.7% of the participants exhibited inadequate or problematic food literacy. After completion of the program, the proportion of participants with adequate food literacy increased significantly (Table 3).

There were no differences between the participants after completing the training programme with regard to gender (Mann–Whitney U test = 72.5, *p* = 0.95) and marital status (Kruskal–Wallis H test = 0.2, *p* = 0.64).

Age and years of work experience were moderately and positively associated with higher literacy levels, and this relationship was statistically significant (*ρ* = 0.5, *p* = 0.01; *ρ* = 0.6, *p* < 0.001, respectively) (Table 4).

## 4. Discussion

This study’s main objective was to evaluate the effectiveness of a training model aimed at promoting food literacy in a rural Angolan community. The results showed a statistically significant increase in participants’ food literacy after the intervention, with adequate levels rising from 13.3% to 73.3% (*p* < 0.001). This improvement is reflected not only in the acquisition of knowledge but also in the development of practical skills related to the selection, preparation, and combination of local foods. These topics were addressed during the training and included topics such as breastfeeding, artificial feeding, dietary diversification, vitamin supplementation, intolerances and allergies, as well as nutrition for children up to six years of age, always adapted to the cultural context and food resources available in Gungo. These results confirm the relevance of promoting health literacy as an effective strategy for mitigating child malnutrition in vulnerable contexts by strengthening the skills of health promoters [6,11]. The relevance of these data is even more pronounced when compared with the inadequate feeding practices widely documented in Angola [12]. The observational study conducted by Pietravalle et al. demonstrated that 25.2% of children aged 6 to 23 months in the province of Cunene presented global acute malnutrition [11]. Similarly, multicentre research by Yonezawa et al. [3] confirmed that the introduction of complementary foods varies substantially between countries, with cereals predominating in Asia and Africa, mainly for reasons of tradition or availability. However, excessively monotonous diets can result in deficiencies in essential micronutrients. This phenomenon was also highlighted by Bottin et al. [13], who concluded that food insecurity and maternal diet directly influence the nutritional composition of breast milk.

The results also demonstrate that age and years of work experience are moderately and positively associated with higher literacy levels, and this relationship is statistically significant. However, this finding is not corroborated by other studies [14], in which evidence suggests that younger adults tend to have higher scores than older adults. Educational level, on the other hand, has a significantly stronger association with better food literacy levels [15].

The intervention implemented in Gungo was designed to address the shortage of specialised services and promote sustained changes in health behaviours, adapting the pedagogical content to local practices and cultural references. Its relevance is reinforced by the sociodemographic data of the sample, which reveal a high proportion of displaced people (80%) and low levels of education, conditions that limit the adoption of adequate nutritional practices. In these contexts, training community health workers constitutes a strategic and sustainable alternative for disseminating good dietary practices [6]. The integration of international evidence [3], national epidemiological data [4], hospital-based clinical studies [6], and community experiences such as the Seigungo project allows for the development of more integrated, culturally appropriate, and effective strategies to address child malnutrition in Angola. Data from the MuCCUA study, conducted by Custodio et al. [16], corroborate this view, demonstrating that multisectoral, nutrition-sensitive interventions, such as nutrition education, are particularly effective when implemented in the first two years of life. Similarly, the results of Moya-Alvarez et al.’s study [17] on vitamin C deficiency in mothers and infants in Central Africa reinforce the idea that insufficient food literacy compromises not only nutritional status, but also immunity and resistance to infections. Thus, the mere availability of food does not guarantee adequate nutrition: access to information and knowledge is a critical determinant of dietary quality. The evidence obtained in this study demonstrates that culturally contextualised and evidence-based training programs can have a significant impact on improving food literacy, especially in rural areas marked by a shortage of qualified human resources and high rates of child malnutrition. The promotion of food literacy should, therefore, be integrated into public health, education, and community development policies. More than simply imparting knowledge about nutrition, it is necessary to develop practical and critical skills that enable individuals to manage complex food environments, also considering social determinants such as poverty, education, and access to healthy foods.

The relevance of these results goes beyond the statistical sphere, also assuming social and political significance. Replicability of this model in other rural Angolan contexts could contribute to the structural reduction of child malnutrition rates and the promotion of equitable access to health knowledge, in line with international development agendas [1,2].

### Limitations and Suggestions

This study’s limitations include the fact that the instrument used (Table 1) has not yet been formally validated. It underwent a process of theoretical construction and cultural adaptation, and in this paper only an internal consistency study was carried out (using Cronbach’s alpha coefficient). Future studies should focus on formal validation of the instrument and the study of other psychometric properties (e.g., validity, reliability, and responsiveness, among others).

Other methodological limitations should be acknowledged. The study’s small sample size and the use of a non-probability sampling method, which limited participant selection to a specific group, constrain the generalisability of its results. On the other hand, the evaluation relied solely on self-reported knowledge and did not include objective indicators of behavioural change or evidence of anthropometric impact on children in the community, which constitutes a significant limitation in measuring the program’s medium- and long-term effectiveness.

## 5. Conclusions

Based on the results obtained, it is verified that the intervention program created by the research team and implemented in the Gungo community was successful. The food literacy module addressed topics such as nutrition education and child malnutrition, demonstrating that, beyond food availability, food literacy is essential for achieving adequate nutrition. The intervention proved feasible, accessible, and replicable, particularly in vulnerable contexts with minimal resources and a lack of qualified health practitioners. Increasing the accessibility and use of similar initiatives that emphasise nutrition education and engage local and community health promoters may significantly improve health outcomes, particularly in environments with limited resources.

## Figures and Tables

**Table 1:** Food literacy scale.

Is It Easy Or Difficult	1	2	3	4	5
1. To find real information about food.					
2. To understand information about healthy eating.					
3. To find information about the daily frequency of meals.					
4. To understand the importance of eating several times a day.					
5. To eat the recommended meals throughout the day, e.g., always taking a snack.					
6. To practise eating in accordance with the principles of healthy eating.					
7. To change your diet to one that is suitable for certain diseases/intolerances.					
8. To use the information you receive to confirm that you are eating well in your daily practice.					
9. To understand the information contained in the Food Wheel.					
10. To understand the warnings about the consumption of sugary/refreshing drinks.					
11. To understand the information on energising drinks (like Red Bull).					
12. To find information on the use of salt in food.					
13. To understand the risks of using too much salt in food.					
14. To find information on sources of animal and vegetable protein.					

**Table 2:** Sociodemographic background.

Variable	Number of Participants		Percentage	
Sex				
Male	18		60	
Female	12		40	
Marital status				
Single	16		53.3	
Married or cohabitating couples	14		46.7	
Level of education				
Primary education				
3 years	1		3.3	
4 years	2		6.7	
6 years	8		26.7	
Secondary education I cycle				
8 years	4		13.3	
9 years	4		13.3	
Secondary education II cycle				
11 years	2		6.7	
12 years	2		6.7	
13 years	2		6.7	
Higher education				
Bachelor’s degree	5		16.7	
Displaced from usual residence				
Yes	24		80	
No	6		20	
Household				
Living alone	2		6.7	
Living with the family of origin	6		20	
Living with other family members	21		70	
Other	1		3.3	
	M	SD	Min	Max
Age	45.5	10.9	24	64
Years of work experience	13.8	8.5	1	36

M: mean; SD: Standard deviation.

**Table 3:** Levels of food literacy.

	Prior to Attending the Training Program		After Completing the Training Program		*p*
	n	%	n	%	
Inadequate	20	66.7	3	10	
Problematic	6	20	5	16.7	
Adequate	4	13.3	22	73.3	
Total score	M = 6.8; SD = 4.07		M = 12.77; SD = 2.07		<0.001

M: mean; SD: Standard deviation.

**Table 4:** Spearman’s rank correlation coefficients.

	Age		Years of Work Experience	
	*ρ*	*p*	*ρ*	*p*
Levels of literacy after completing the training program	0.5	0.01	0.6	<0.001

*ρ*: Spearman’s rank correlation; *p*: *p*-value.

## Data Availability

Data are available upon reasonable request.
